# Combined linkage and family-based association analysis improves candidate gene detection in Genetic Analysis Workshop 18 simulation data

**DOI:** 10.1186/1753-6561-8-S1-S29

**Published:** 2014-06-17

**Authors:** Yi Li, Jia Nee Foo, Herty Liany, Hui-Qi Low, Jianjun Liu

**Affiliations:** 1 Human Genetics, Genome Institute of Singapore, #02-01, Genome, 60 Biopolis Street, 138672, Republic of Singapore

## Abstract

Because the genotype-phenotype correlation information is investigated differently by linkage and association analyses, various efforts have been made to model linkage and association jointly. However, joint modeling methods are usually computationally intensive; hence they cannot currently accommodate large pedigrees with dense markers. This article proposes a simple method to combine the linkage and association evidence with the aim of improving the detection power of disease susceptibility genes. Our detection power comparisons show that the combined linkage-association *p *values can improve remarkably the causal gene detection power in Genetic Analysis Workshop 18 simulation data.

## Background

Linkage analysis in family data looks for the genomic region where the disease phenotype of interest and a stretch of genetic markers are cosegregated. As a result of the strong identity-by-descent (IBD) sharing among family members and a limited number of recombination events present in collected pedigrees, the critical regions detected by linkage analyses rarely pinpoint a single gene. However, linkage analysis is immune to the confounding of population stratification suffered by association analyses. Association analyses regress quantitative phenotypes on a marker's genotypes or compare allele frequencies of a single-nucleotide polymorphism (SNP) between cases and controls, and can narrow down the putative disease regions to small regions of high linkage disequilibrium (LD blocks), which are usually much shorter than linked regions. With the advance of next-generation sequencing technology and highly accurate imputation methods, association analyses with dense marker coverage can even potentially locate candidate causal variants (and thus candidate genes) directly. Because the genotype-phenotype correlation information is investigated differently by linkage and family-based association analyses, various efforts have been made to model linkage and association jointly [1-9]. Naming a few among many, Li et al [6] proposed 2 likelihood ratio tests in a joint linkage-association model to characterize whether an associated SNP can partially or completely explain linkage signals; Goring and Terwilliger [4] proposed a joint linkage and LD model through the use of a pseudomarker locus. Joint modeling methods [1,3-6] are usually computationally intensive; hence they cannot currently accommodate large pedigrees with dense markers. This article proposes a simple method to combine the linkage and association evidence with the aim of improving the detection power of disease susceptibility genes. Specifically, we convert the linkage LOD score to *p *values and adopt the unweighted Liptak [10] method to combine the linkage and association *p *values. Our detection power comparisons show that the combined linkage-association *p *values can improve the causal gene detection power remarkably in Genetic Analysis Workshop 18 (GAW18) simulation data.

All the analyses and comparisons in this report are performed with the disease causal variants known.

## Methods

### Long-term mean blood pressure

We adopt the method found in Levy et al [11] to adjust for the effects of age, sex, and medication status on the blood pressure, and calculate the long-term mean systolic blood pressure (SBP) on the basis of the 3 time-point-adjusted SBP measurements.

### Multipoint quantitative trait linkage analysis (SOLAR)

SOLAR [12] is a variance component multipoint linkage analysis software for quantitative traits. In the restricted model, the additive genetic variance because of the quantitative trait locus (QTL) of interest equals zero, whereas in the alternative model the additive genetic variance because of the QTL of interest is estimated by maximizing the likelihood of the model. The linkage LOD score is the difference log_10 _in likelihood between the alternative and the restricted models. A total of 3071 genome-wide association studies (GWAS) array SNPs were randomly selected so that they were not in high LD in unrelated individuals. Multipoint linkage analysis in SOLAR [12] was applied to the LD-pruned SNPs on the quantitative traits Q1 and mean SBP.

### Family-based association test using multiple markers

The multimarker version of family-based association test (FBAT) statistics is a linear combination of single-marker FBAT statistics with the data-driven combination weights [13]. We adopt the option -e in the FBAT package, which forces it to estimate the association signal in the presence of linkage. The analysis unit is a gene whose starting and ending physical positions are obtained from the UCSC refgene database. The imputed genotypes of all the nonsynonymous SNPs in a gene were analyzed together to obtain gene-based association *p *values.

### Combining linkage and association evidence

In the output from SOLAR, LOD scores were given with respect to genetic distances; the physical boundaries for each gene were mapped to genetic distances, and a gene was assigned the average LOD score of the genetic region to which it is mapped. Next, the linkage LOD score is converted to a *p *value by observing that 2*log_*e*_(10^LOD^) is asymptotically distributed as a 0.5:0.5 mixture of a $\chi_{1}^{2}$ variable and a point mass at zero [12]. The linkage and association *p *values for a gene are inverse-normal transformed to *Z*_1 _and *Z*_2 _respectively. We then adopt the following unweighted Liptak method [10] to combine linkage and association evidence and obtain a combined *p *value. When *Z*_1 _and *Z*_2 _are independent, $Z_{c}{\text{ = l}}_{k}^{T}{\left(Z_{1},Z_{2}\right)}^{T}/\sqrt{{\text{l}}_{k}^{T}\Phi {\text{l}}_{k}}$ where l_*k *_is a k-element vector of 1, Φ is a 2 × 2 identity matrix, and (*Z*_1 _,*Z*_2 _) is a row vector made up of *Z*_1 _and *Z*_2 _that follows the standard normal distribution asymptotically. When *Z*_1 _and *Z*_2 _are correlated [14], Φ can be empirically estimated as the correlation matrix of the matrix $P=\left(Z_{1}^{b},Z_{2}^{b}\right)$, where $Z_{j}^{b}$ (*j *= 1,2) is an *N*-element column vector of test statistics for test *j *when the phenotypes are permuted *N *times. The combined linkage and association *p *values were calculated using Liptak method with and without correlation correction.

## Results

The linkage analysis showed that chromosome 3 had an LOD score >1.5 three and nine times among simulations 1 to 10 for the traits of Q1 and mean SBP, respectively. Most of the linkage regions for the trait of mean SBP were mapped around 55 to 70 cM, whereas for the trait of Q1, the linkage regions were quite scattered, being0 to 30 cM, 125 cM, and 165 to 220 cM for the 3 simulations with LOD scores >1.5. It turned out that chromosome 3 had the strongest linkage signal.

FBAT was applied to 8047 genes among 11 chromosomes that have more than 1 nonsynonymous SNP. We mimicked the fast validation strategy in practice, which took top 50 candidates to validate in independent samples. Because we investigated gene-based analyses, we took a *p *value threshold so that top 50 genes were checked against the simulated disease model. For mean SBP, on average, 49 of 8047 genes had combined *p *values less than 0.001 among simulations 1 to 10. Only 2 causal genes, *MAP4 *and *FLNB *on chromosome 3, were ever among the top 49, so we investigated their detection power. For Q1, on average, there were 9.5 and 9.1 genes out of 8047 with FBAT *p *values and combined *p *values smaller than 0.001, corresponding to an empirical false-positive rate of 0.0012 and 0.0011, respectively.

Although the combined *p *values were slightly different when the correlation between linkage and association *p *values was corrected, the ranks of these 2 genes (out of 8047) based on the combined *p *values did not change. Table 1 shows the ranks of the 2 causal genes based on the association *p *values and the combined *p *values for the traits Q1 and mean SBP.

**Table 1:** Ranks of 2 causal genes (*MAP4 *and *FLNB*) for trait Q1 and mean SBP based on FBAT *p *values and combined linkage and association *p *values for simulations 1 to 10.

		S1	S2	S3	S4	S5	S6	S7	S8	S9	S10
**MAP4**	Q1	346	3490	1645	3123	1296	2870	976	4890	3103	733
	
	combined Q1	920	1073	2429	4310	1651	4105	1353	5142	3958	1816
	
	mean SBP	10	**201**	**479**	**55**	**86**	9	18	**154**	3	35
	
	combined mean SBP	7	**12**	**12**	**3**	**5**	3	4	**6**	2	3

**FLNB**	Q1	3545	2914	4074	2836	5322	3273	4499	1334	3276	4586
	
	combined Q1	4193	3326	4568	4090	4132	4355	4717	1939	3958	4690
	
	mean SBP	681	5660	335	**317**	**905**	**668**	590	2372	**104**	5674
	
	combined mean SBP	266	1084	144	**9**	**28**	**10**	240	343	**10**	517

Useful rank improvements are in bold fonts.

For the trait of mean SBP, the combined *p *values were viewed to improve the FBAT *p *values if the rank of the causal gene based on the latter was beyond 49, and the rank based on the former was within 49. There were 5 and 4 improvements for *MAP4 *and *FLNB*, respectively (highlighted in Table 1). On the contrary, there was no such improvement for the trait Q1.

## Discussion

Generally speaking, the power for detecting the causal genes was low, except for *MAP4*, which explains a large percentage of SBP variance (7.79%). Combined *p *values improved the detection power for *MAP4 *from 50% to 100%. For *FLNB *that explains a much lower percentage of SBP variance (0.29%); FBAT had no detection power. Combined *p *values improved the power to 40%. Moreover, the type I error was well controlled in our combined *p *values. These results indicated a promising strategy of combining the linkage and association evidence to improve the true discovery rate/power. Furthermore, our method combines the linkage and association *p *values in a simple way; thus it is applicable to large pedigrees as long as large pedigrees can be accommodated in the linkage analyses. The option -e in FBAT software forces an estimation of association in the presence of linkage, thus the association signal detected is expected to be independent of the linkage signal. That the combined *p *values with and without correlation correction were very similar (correlation coefficient >0.99, data not shown) verified this.

The combined *p *values we propose to calculate depend on the strength of both linkage and association signals. Moderate signals in both linkage and association will generate a more significant combined *p *value than a significant signal in one test but a null signal in the other. To maximize the association power, we analyzed only nonsynonymous SNPs in gene-based association tests, as we know that the nonsynonymous SNPs are enriched with causal variants with relatively large effects from the released disease model. In real sequencing projects, especially whole genome sequencing studies, we may select other functional variants to analyze, such as deleterious or regulatory SNPs, to improve the association power.

In our opinion, the combined test is more powerful because linkage and association analyses investigate different parts of phenotype-genotype correlation, thus providing nonredundant information. Combining these 2 *p *values makes some causal genes that have moderate supports in both tests stand out. For example, for simulation 8, chromosome 3 had a LOD score of <1.5. However, the regions to which *MAP4 *and *FLNB *were mapped still have moderate linkage evidence, with LOD scores of 0.82 and 0.53, respectively. As a result, the ranks improved from 154 (FBAT *p *value = 0.0137) to 6 (combined *p *value = 0.00166) for *MAP4*, and from 2372 (FBAT *p *value = 0.195) to 343 (combined *p *value = 0.0430) for *FLNB*.

## Conclusions

We proposed a simple method to combine the linkage and family-based association evidence that is applicable to large pedigrees. Our results showed that the combined linkage and FBAT *p *values do improve the causal gene detection power remarkably. The improved true discovery will render a higher chance for the top genes to be validated.

## Competing interests

The authors declare that they have no competing interests.

## Authors' contributions

YL and JL conceived of the study and its design, YL analyzed the data and wrote the manuscript. JNF helped with the long-term mean SBP calculation; HQL and HL processed the genotype data. All authors read and approved the final manuscript.
